# Oscillatory Dynamics Supporting Semantic Cognition: MEG Evidence for the Contribution of the Anterior Temporal Lobe Hub and Modality-Specific Spokes

**DOI:** 10.1371/journal.pone.0169269

**Published:** 2017-01-11

**Authors:** Giovanna Mollo, Piers L. Cornelissen, Rebecca E. Millman, Andrew W. Ellis, Elizabeth Jefferies

**Affiliations:** 1 Department of Psychology, University of York, York, United Kingdom; 2 Department of Psychology, School of Life Sciences, Northumbria University, Newcastle upon Tyne, United Kingdom; 3 York Neuroimaging Centre, University of York, York Science Park, York, United Kingdom; 4 Audiology and Deafness Group, School of Psychological Sciences, University of Manchester, Manchester, United Kingdom; Universita degli Studi di Udine, ITALY

## Abstract

The “hub and spoke model” of semantic representation suggests that the multimodal features of objects are drawn together by an anterior temporal lobe (ATL) “hub”, while modality-specific “spokes” capture perceptual/action features. However, relatively little is known about how these components are recruited through time to support object identification. We used magnetoencephalography to measure neural oscillations within left ATL, lateral fusiform cortex (FC) and central sulcus (CS) during word-picture matching at different levels of specificity (employing superordinate vs. specific labels) for different categories (manmade vs. animal). This allowed us to determine (i) when each site was sensitive to semantic category and (ii) whether this was modulated by task demands. In ATL, there were two phases of response: from around 100 ms post-stimulus there were phasic bursts of low gamma activity resulting in *reductions* in oscillatory power, relative to a baseline period, that were modulated by both category and specificity; this was followed by more sustained power decreases across frequency bands from 250 ms onwards. In the spokes, initial power *increases* were not stronger for specific identification, while later power *decreases* were stronger for specific-level identification in FC for animals and in CS for manmade objects (from around 150 ms and 200 ms, respectively). These data are inconsistent with a temporal sequence in which early sensory-motor activity is followed by later retrieval in ATL. Instead, knowledge emerges from the rapid recruitment of both hub and spokes, with early specificity and category effects in the ATL hub. The balance between these components depends on semantic category and task, with visual cortex playing a greater role in the fine-grained identification of animals and motor cortex contributing to the identification of tools.

## Introduction

Conceptual processing plays a crucial role in our lives, allowing us to understand the significance of words and objects and to guide our behaviour accordingly [1, 2]. However, the question of how conceptual knowledge is represented and retrieved remains controversial, with different theories and research methods variously suggesting a crucial role for (i) an anterior temporal lobe (ATL) ‘hub’ across categories and modalities [3–5] and (ii) modality-specific perceptual and motor regions of cortex (‘spokes’), reflecting the visual, auditory and action features of the concept being retrieved [6–8]. Since both these components are engaged during conceptual retrieval, it becomes important to consider (i) *when* hub and spoke regions are engaged following the presentation of a stimulus and (ii) *how* their recruitment is modulated by task demands–including the type of object to be identified (i.e., animal vs. manmade object) and the level of identification required (i.e., coarse- vs. fine-grained identification). This study uses magnetoencephalography (MEG) to address these questions. The view that knowledge is captured in the links between different motor and sensory representations is supported by a wealth of neuroimaging studies that have shown differential patterns of activation for concepts that draw on different types of features: thinking of a rose produces activation in cortical regions linked to colour and smell processing (alongside other regions), while thinking of a tennis racquet elicits additional areas of activity in regions linked to action and praxis [6, 9–11]. This principle may underpin category-specific effects in conceptual processing, since visual and motor/praxis features are likely to be important for differentiating animals and manipulable manmade objects respectively [12–14]. Animals are visually complex yet have highly overlapping visual features (e.g., four legs, tails, eyes, ears)–thus specific visual features are important in differentiating one animal concept from another, e.g., the stripes on a zebra distinguish it from a horse [15, 16]. Manmade objects have more diverse visual features at the superordinate level, and thus might not show the same interaction between visual processing and specificity [17–20], instead, when artefacts must be identified as a ‘nut-cracker’ or a ‘knife’, the different actions and grips associated with these objects may be crucial for distinguishing them [17, 21–23].

Following the presentation of words denoting action concepts, activation within motor cortex occurs rapidly (within 150ms): activity of the motor hand area is seen for words such as “pick”, while the leg area shows activation for “kick” [24–26]. Given this rapid activation, links between words and their motor/perceptual referents are likely to play an important role in accessing meanings [7]; however, the recruitment of motor ‘spokes’ is also modulated by their relevance to the task [27–29]. In addition, similarities in any given sensory/motor region do not always predict deeper semantic relationships [1, 3]: for example, a kiwi and a banana are highly semantically related, and yet they have different verbal labels, colours, shapes, textures and require different actions to peel the skin. Consequently, sensory-motor links may be supplemented by an amodal ‘hub’ in the ATLs, allowing mappings between modalities and the extraction of deep semantic similarities based on the sum of all of our experiences with objects and words [1]. This hypothesis was motivated by studies of semantic dementia (SD), a condition characterised by bilateral ATL atrophy and hypometabolism plus progressive deterioration of knowledge which proceeds in a fine- to course-grained fashion. These patients show more impaired naming and matching for specific than superordinate labels (e.g., Dalmatian vs. animal) [1, 30–33]. This is purportedly observed because objects with highly overlapping features–e.g., horse and zebra–are represented by similar patterns of ATL activity which become indistinguishable as the semantic representations degrade [34, 35]. Neuroimaging studies also show greater ATL activation for specific as opposed to superordinate judgements [18, 36], while inhibitory TMS to this region disrupts specific not superordinate picture naming [37].

Thus, both hub and spoke regions appear to be necessary for the efficient retrieval of conceptual knowledge [37, 38]. However, little is known about when and how these distinct components are recruited. The traditional view is that semantic access occurs around 400ms post-stimulus onset, since EEG studies show a highly reliably reduced negative component in this time window (N400) when the target meaning is semantically primed [39, 40]. Vartiainen and colleagues [41] and Lau and colleagues [42] reported similar effects from 300–500 ms in superior temporal areas using MEG. Nevertheless, there is building evidence for much more rapid engagement of ATL in visual object recognition and verbal semantic tasks, as documented in several recent electrophysiological studies [43–51]. For example, stronger co-activation of ATL and visual cortex was observed around 150 ms post stimulus onset in a picture naming task for basic-level compared to superordinate name retrieval and for living vs. non-living items [45]. This work helps to uncover the time-course of the interaction between the ATL hub and a visual ‘spoke’ although it is not yet known whether other ‘spoke’ regions (e.g., motor cortex) interact with ATL in a similar way.

We addressed this issue by using time-resolved MEG methods to examine the predictions of the hub and spoke framework. We measured neural oscillations within left ATL (the ‘hub’ site) and two distinct ‘spoke’ regions; one in posterior lateral fusiform cortex that should contribute to the representation of visual features (FC; visual spoke) and one in central sulcus close to the motor and somatosensory hand areas (CS; motor/somatosensory spoke) that should contribute to the representation of motor/praxis features. We examined the engagement of these regions during a word-picture matching paradigm that required participants to map between verbal and pictorial representations of the same object, given that this is hypothesised to be a key function of the ATL hub. Within this task, we varied both semantic category (manmade vs. animal) and the specificity of identification required (corkscrew vs. manmade; Dalmatian vs. animal). This allowed us to test specific hypotheses about the contribution of the ATL hub and spokes to the identification of animals and manmade objects through time. The hub-and-spokes account draws on the principle of interactive-activation and anticipates that the *simultaneous* activation of these components underpins semantic processing. Consequently, we might envisage that effects of specificity should emerge in *both* the hub and spokes at a similar time and at a relatively early stage (i.e., within the first 200ms, as opposed to these effects only emerging around 400ms post-stimulus–i.e., in the N400 window). Alternative accounts envisage feed-forward activation from the visual spoke to the ATL hub–and consequently effects of specificity could occur earlier in visual cortex than ATL (which might not show effects of specificity until around 400ms post-stimulus)[52].

The hub-and-spokes model also predicts that the importance of visual and motor spoke regions to conceptual identification will depend on semantic category. We might expect identification of animals and manmade objects to show a dissociable response across visual and motor cortex, since motor features should be particularly important for the identification of manmade objects with associated actions, while visual features are likely to be important in a visually-presented word-picture matching paradigm for both animals and manipulable manmade objects. Moreover, non-shared visual features such as ‘stripes’ might help to differentiate *specific* animals (such as zebra) from other animals with overlapping features (horse); therefore, we would expect a double-dissociation in the recruitment of motor and visual ‘spokes’ for specific-level categorisation–i.e., visual regions should make a greater contribution to the identification of specific animals, while motor regions are more crucial for tools. Again, the hub-and-spokes framework anticipates that any effects of semantic category should occur at a similar point in time in the hub and spokes, and at a relatively early time point (e.g., within the first 200ms, as opposed to 400ms post-stimulus), since it is the simultaneous recruitment of hub and spoke regions that should give rise to semantic category effects. Again, alternative accounts based on feed-forward activation from visual cortex to ATL might envisage effects of semantic category in visual regions that precede the emergence of these effects in ATL.

We employed time-frequency (TF) analyses to characterize task-related oscillatory changes in hub and spokes, since this approach is a powerful tool for understanding the emergence of cognitive processes. The majority of MEG and EEG studies to date have examined semantic processing in the time-domain (e.g., evoked potentials) and have focused on the phase-locked components of oscillatory power. In this study, we instead considered the frequency domain, and examined total power, which has both phase-locked and non-phase locked signal components. Total power is a suitable dependent measure given our research questions, as it is sensitive to effects in complex tasks when the phase relationship to the input is not expected to be preserved across participants or trials. Changes in total power below 50 Hz have already been associated with the retrieval of semantic features in memory and language tasks [51, 53, 54]. In ATL, increased theta power has been associated with cross-modal integration of lexical-semantic information [55], while power changes in alpha and beta frequencies are linked to object recognition [51]. In addition, oscillatory activity in visual regions has been shown to be modulated by the semantic properties of words and pictures [51, 53]. The current study builds on work showing the co-recruitment of visual regions and ATL object naming at the specific level, to characterise the recruitment of visual and motor spoke regions alongside the ATL hub during specific and superordinate categorisation of animals and manmade objects. This allowed us to test the predicted effects of specificity and category at these sites, and to characterise the similarities and differences of hub and spoke responses in time and frequency.

## Material and Methods

### Participants

Twenty-six healthy volunteers participated in the MEG experiment (8 males, mean age 24, 19–34 years) and sixteen participants (5 males, mean age 24, 18–34 years) took part in a separate behavioural experiment. All participants were native English speakers, right handed, had normal vision and reported no history of language disorders, neurological or psychiatric illness. The current study was approved by the Research Ethics and Governance Committee of the York Neuroimaging Centre, University of York, UK, and written informed consent was obtained from all participants. Six MEG datasets were excluded from analysis due to excessive movements or artefacts (see data acquisition and pre-processing for details).

### Experimental design and procedures

**Fig 1:**
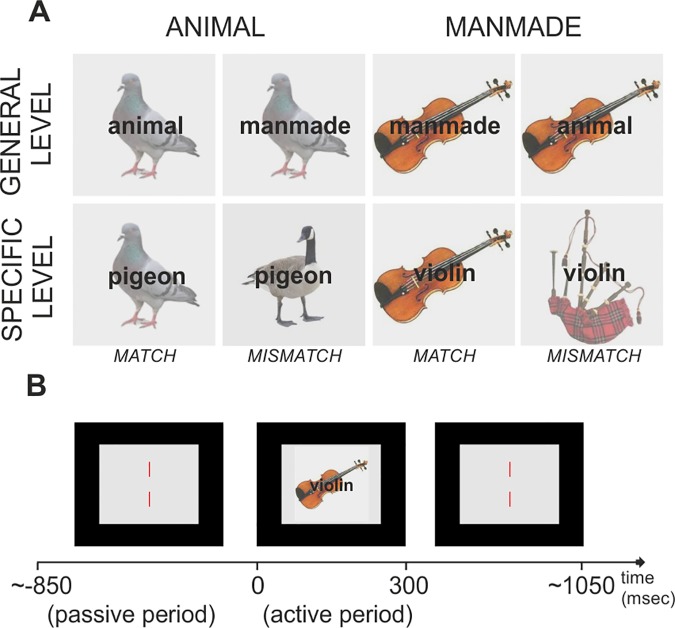
(a) Experimental Design. We used a word-picture verification task in which pictures of animals or manmade objects were identified as members of either these superordinate categories, or at a more specific level. (b) Trial Structure. Words and pictures were presented simultaneously and participants were asked to press a button with their left hand when the picture and word did not match. These mismatching trials requiring an overt response occurred on 30% of trials and were not included in the subsequent MEG analysis.

This experiment employed a word-picture verification task in which pictures of items from two semantic categories (*animals*–e.g., zebra and *manipulable manmade objects*–e.g., screwdriver) were identified as members of either these *general* categories, or at a more *specific* level (i.e., using their specific names zebra and screwdriver). This gave a 2X2 design in which semantic category determined the relevance of visual and action features to identification, while superordinate- and specific-level trials were compared to manipulate the importance of accessing detailed visual and motor features (cf. [45]). The verbal label (e.g., ‘animal’ or ‘Dalmatian’) defined the level of processing for the target concept. Each condition comprised 120 trials where the word was congruent with the picture; these 480 matching trials were pseudo-randomly intermixed with 180 trials (45 trials per condition) where the word and picture did not match. Examples of matching and mismatching trials are shown in Fig 1.

We used simultaneous presentation of words and pictures, in order to provide a clear onset for semantic retrieval. Similar paradigms have been extensively studied behaviourally, with data suggesting that word processing is faster and dominant over picture processing [56–59]. For example, the picture-word interference paradigm presents words superimposed on pictures and shows that word reading is relatively immune to the simultaneous presentation of semantically related pictures, while semantically-related words influence picture naming [59]. Therefore, in our paradigm, we can be confident that processing the semantic content of pictures is influenced by the level of specificity defined by the words presented on each trial.

The stimuli used for the experiment consisted of colour photographs of 60 animals and 60 manmade manipulable objects, each of which was presented twice using two different images of the same object. These images were selected from a large dataset comprising at least three different images of the same object. Ratings of different semantic and psycholinguistic characteristics of these concepts/images were also collected using separate online surveys employing five independent groups of participants (not included in the MEG study). On 5-point Likert scales, participants rated how much a given picture was a good fit to a particular concept (*Image Agreement*), the extent to which that concept was representative of the category to which it belongs (animal or manmade object) *(Typicality)*, how familiar the concept was *(Familiarity)* and the extent to which visual, action and visual-motor features defined the concept *(Semantic Features)*. Finally, for the two pictures of each concept with the higher image agreement score, we assessed how consistently each picture was named at the most specific level *(Concept Agreement)* and used those labels for the specific-level condition. The lists of words used for defining the picture at specific level were matched between conditions in terms of number of letters (p = .84) and lexical frequency (p = .71) obtained from the SUBTLEX_UK database [60](for details see S1 Table). Similarly, the stimuli used for the two conditions were matched for image agreement (p = .20), name agreement (p = .35) and familiarity (p = .23), but also for the number of non-white pixels as a measure of image complexity [61](p = .72). The items in the Animal and Manmade conditions were significantly different in terms of their predominant semantic features: animals scored more highly for visual (p < .001) and visual-motion features (p < .001), whereas manmade objects scored more highly for action features (p < .001) (see S1 Table for details). The two lists also differed for typicality (p = .01), because animals were generally rated as more typical members of the superordinate category than manmade objects (animal = 4.18, manmade object = 3.86); this is likely to reflect the greater similarity between the features of animals than manmade objects. The colour photographs of the animals and manmade objects selected for the MEG experiment were presented on a back projection screen (60Hz) in a dark room. Images appeared in a 14.4° × 14.4° region in the centre of the screen (when viewed from the standard distance of 60cm) which was set to a mid-grey level. The average luminance of the stimulus display region was in the mesopic range. As illustrated in Fig 1, a pair of red nonius lines was presented in the same mid-grey region, helping participants to maintain a steady fixation. The nonius lines were present throughout each experimental run, except when replaced briefly by stimulus pictures which appeared in the middle of the patch for 300ms. The contrast in the stimulus pictures was reduced sufficiently to ensure that the superimposed dark grey text was easily visible (Arial Monospace). Each letter subtended ~0.75° horizontally.

Each trial started with the nonius red lines. After a variable interval of 700–1000 ms, the target was projected onto the screen for 300 ms. The inter-trial interval (ITI) varied between 900 and 1200 ms, and this was increased by 3000 ms in the event of a button press in the MEG version of the experiment. The behavioural pilot of this experiment required the participants to use the two mouse buttons to indicate whether the compound picture-word either matched or not, while in the MEG experiment, participants were asked to press a button with their left index finger only when picture and word did not match, and these trials were excluded from further analysis of the MEG data. The experiment was controlled using Presentation 16.1 (Neurobehavioral Systems). To familiarize participants with the task, 20 practice trials were performed at the beginning of the experiment but they were not included in the analysis. For each participant, the experiment was administered in six blocks of approximately 7 minutes each, separated by self-paced breaks. Block order was randomized across subjects. For the MEG experiment, participants were instructed to keep still throughout the experiment, and to avoid any movement not related to the task. They were asked to blink only after making a button press.

### Data acquisition and pre-processing

Before MEG data acquisition, participants’ head shape and the location of five head coils were recorded with a 3D digitizer (Fastrak Polhemus). The signal from the head coils was used to localise participant’s head position within the helmet before and after the experiment. For each participant, a high-resolution structural T1-weighted anatomical volume was acquired in a GE 3.0 T Signa Excite HDx system (General Electric, USA) at the York Neuroimaging Centre, University of York. The 3D digitized head shape of each participant was used for the co-registration of individual MEG data onto the participant’s structural MRI image using a surface-based alignment procedure [62].

MEG data were collected in a magnetically shielded room using a whole-head 248-channel, Magnes 3600 (4D Neuroimaging, San Diego, California), with the magnetometers arranged in a helmet shaped array. Data were recorded in continuous mode, with a sampling rate of 678.17 Hz and pass-band filtered between 1–200 Hz. MEG signals were subjected to a global field noise filter subtracting external, non-biological noise detected by the MEG reference channels, and converted into epochs of 1300 ms length, starting 500 ms before the target onset. Mismatch trials were discarded from any functional analysis. Each epoch was visually checked and excluded from further analysis in the event of response errors and/or artefacts, such as eye blinks, other movements, or electrical noise. Statistical analyses included only datasets with at least 75% of trials retained after artefact rejection. Twenty datasets reached this criterion. On average, 12% of the trials were rejected from these datasets (min 5%—max 25%).

### Analysis strategy

The spatial and temporal resolution of the MEG recordings was exploited in a two-step analysis: first, we examined the response of the whole brain to the task (across conditions) and (in a supplementary analysis) to the main effects of specificity and semantic category, at a coarse frequency resolution and averaging out the temporal component. Secondly, we interrogated the activity of specific cortical regions engaged by the task at a finer frequency and temporal scale.

For both analyses, the neural sources of the brain activity were reconstructed with a modified version of the vectorised, linearly-constrained minimum-variance (LCMV) beamformer described by Van Veen et al, 1997 [63] and referred by Huang et al., 2004 [64] as Type I beamformer, implemented in the Neuroimaging Analysis Framework pipeline (NAF, York Neuroimaging Centre), using a multiple spheres head model [65]. An MEG beamformer (spatial filter) allows an estimation of the signal coming from a location of interest while attenuating the signal coming from other points in the brain. This is achieved by constructing the neuronal signal at a given point in the brain as the weighted sum of the signals recorded by the MEG sensors. Independent beamformers were reconstructed for each point in the brain, in each of three orthogonal current directions, separately. In our analysis, the covariance matrix used to generate the weights of each beamformer was regularized using an estimate of noise covariance as described in Prendergast et al., [66] and Hymers et al., [67]. This procedure was performed separately for each condition and/or analysis window, in order to obtain an optimal sensitivity to the effect of interest [68, 69]. The outputs of the three spatial filters at each point in the brain (referred to as a Virtual Electrode) were summed to generate the total oscillatory power, thus combining both phase locked (“evoked”) and non-phase locked (“induced”) signal components [70]. For the whole-brain analysis, a noise normalised volumetric map of source total power was produced over a given temporal window and within pre-specified frequency bands. For the region of interest analysis, the time course information at the location specified was reconstructed and the time-frequency decomposition was computed using Stockwell Transforms [71].

This analysis strategy and the parameters used for the current study were similar to those used in recent MEG studies of visual word recognition and object naming [51, 72, 73]. All information necessary to reproduce these analyses is stated below and the analysis pipeline is also in the public domain (http://vcs.ynic.york.ac.uk/docs/naf/index.html).

### Whole-brain beamforming

The brain’s response to the task was characterised within broad frequency ranges across 500 ms (averaging out the temporal component). The purpose of this analysis was to identify brain regions important for the task in general terms, so that relevant sites could be investigated in more detail in a regions-of-interest analysis (see below).

**Fig 2:**
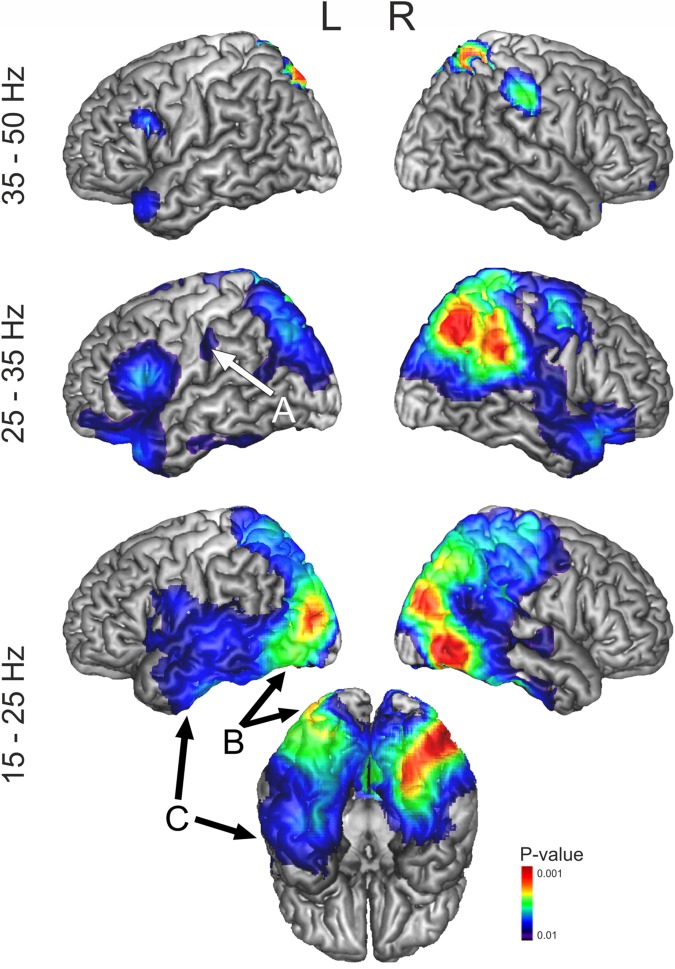
3D rendered cortical representations showing significant activity above baseline across conditions, during 500 ms post-target onset in four frequency bands (15–25 Hz, 25–35 Hz and 35–50 Hz). t-Maps are thresholded at p<0.01 (corrected). All the activations represent event related desynchronization. Significant event related desynchronization was only observed between 5–15 Hz in a region of the right fusiform gyrus that overlapped with the activity observed at 15-25Hz, at a reduced threshold (p = 0.05) and thus this frequency band was omitted from this figure, although all of the whole-brain maps can be accessed from Neurovault (http://neurovault.org/collections/1937/). Arrows indicate the locations selected for the VE analysis.

A 3D lattice of points was constructed across the whole brain with 5-mm spacing, and beamformers were used to compute the total power at each point using the Neural Activity Index (NAI) [63]–an estimate of oscillatory power that takes account of spatially-inhomogeneous noise–at each point independently, within the following frequency pass-bands: 5–15 Hz, 15–25 Hz, 25–35 Hz and 35–50 Hz. Filtering was achieved with 4th order Butterworth filters with automatic padding to eliminate edge artefacts. These frequency ranges represent a subdivision of the frequency spectrum in step of 10Hz (or 15Hz in the case of the gamma band). The frequency bands roughly matched the frequencies of alpha, low and high beta and low gamma band although their purpose was to characterise strong sources of oscillatory power across the whole brain in general terms, to support the selection of point-of-interest to interrogate in the second step of analysis in which we could examine responses across the full range of frequencies in a fine-grained and continuous way. A similar approach was used in previous MEG studies of reading [72, 73], to describe the brain dynamics underlying lexical-semantic processing. We examined total power, which combines evoked (phase-locked to the stimulus) and induced (non-phase locked) components, in each frequency band, comparing an active period (0-500ms following stimulus onset) to a baseline passive period (from -550 to -50 ms before the stimulus was presented). For each individual participant and each frequency band, this analysis produced an NAI volumetric map for the active and passive period. A paired-samples t-statistic was used to characterise the difference between active and passive windows at each point in space in these maps. Individual participant's t-maps were transformed into standardized space and superimposed on the MNI template brain with the cerebellum removed using MRIcroN software [74] (see group t-maps in Fig 2 and S1 Fig).

In order to determine whether the difference between active and passive periods was statistically significant for each point on the lattice, we built up a null distribution by randomly relabelling the two time points for each participant and each voxel, using the permutation procedure developed by Holmes et al. [75]. We established the maximum t-value obtained with random relabelling across 10000 permutations. We then compared the real distribution of t-values in our data with the maximum t-value obtained from the permuted active and passive windows. Maximum statistics can be used to overcome the issue of multiple comparisons (i.e. controlling experiment-wise type I error), since the approach uses the highest permuted t value across the brain to provide a statistical threshold for the whole lattice of points, over which the null hypothesis can be rejected [69]. Fig 2 and S1 Fig show those voxels in the brain with t-values equal or higher than the top 5% or 1% t-values present in the null distribution. We repeated this analysis with all four conditions collapsed together, to characterise the general response of the brain to the task, and also (in a supplementary analysis) examined the main effects of specificity and category, with these conditions being compared with their own passive baseline.

### Time-frequency analysis: points of Interest

Separate beamformers were used to reconstruct the neural activity for three points of interest (POI), in order to characterise the response of these regions to our experimental manipulation over time and frequency with greater precision. The MNI coordinates for these POIs were defined within these pre-specified regions using local peaks of maximum activation across all conditions in the group level, whole brain analysis.

(1) One POI was identified within lateral posterior fusiform gyrus (*FG*, MNI coordinate: -50, -70, -14), since this region is involved in visual object identification [21, 46]. Visual processes within this region should make a critical contribution to the recognition of both animals and man-made objects; however, we would anticipate greater engagement for animals than manmade objects at the specific level, since specific visual features are thought to play a greater role in distinguishing between animals with highly-overlapping visual features [17, 76]. Previous research has linked posterior fusiform to the visual discrimination needed to distinguish between different types of animals which have highly overlapping visual forms (i.e., four legs and a tail) [36, 77]. A similar site showed a greater response for animals than tools in the meta-analysis of Chouinard and Goodale [12]. We would anticipate that this site supports visual aspects of semantic processing.

(2) A second POI was selected within central sulcus (*CS*, MNI coordinate: -54, -22, 42). The motor and somatosensory hand regions to either side of this sulcus have been shown to be activated by tool concepts and their associated hand actions [10, 12, 21–23, 29]. Therefore, for this site, we would expect greater engagement for manipulable manmade objects than for animals if action features are an important component of our conceptual knowledge about tools. While our main analyses focus on putative spokes within visual and motor cortex, the literature on tool semantics suggests two additional sites that could also make a greater contribution to the identification of manipulable manmade objects than animals. First, left premotor cortex is associated with tool and action comprehension [22, 23, 29, 78], although this site is also likely to be influenced by the control demands of semantic tasks [79, 80]. We present results for this site in S2 Fig. Second, left inferior parietal cortex is associated with tool use and hand praxis [22, 78]. We do not present a POI analysis for this location because there was no clear response to the task within this region in the whole-brain beamforming results (see below).

(3) A POI within the anterior inferior temporal lobe (*ATL*, MNI coordinate: -51, 6, -39) was defined using coordinates taken from Binney and colleagues [81]. Atrophy in this region is linked to impaired semantic processing in SD patients, and ATL has been shown to be recruited by semantic tasks across categories in normal participants using distortion-corrected fMRI and transcranial magnetic stimulation [5, 37, 38, 81–83]. Within the whole-brain beamforming data, the ATL response fell within an area of significant activity in the group level analysis, although there was no clear local peak. In order to confirm that the pattern of results observed were not selective to this site, time frequency analysis was also performed on another region within the medial anterior temporal lobe taken from a recent MEG study of visual object recognition [45]. The results from this analysis are reported in S3 Fig.

We elected to examine left-hemisphere sites since (i) fMRI and patient studies reveal a greater contribution of the left hemisphere to semantic processing in general [84]; and (ii) given our participants were right-handed, the motor simulation elicited by single-handed tools was expected to be left-sided. Moreover, right motor cortex might have shown irrelevant responses related to the preparation of button presses with the left hand, even though button presses were only required on mismatching catch trials which were excluded from the analysis.

After the time-series of each POI was reconstructed epoch by epoch, for each subject, by means of separate beamformers [64], time-frequency plots showing total power were computed using Stockwell transforms [71] over a time window from -500 to 800 ms (to avoid edge effects) and a frequency range from 5–50 Hz (frequency resolution 1.33 Hz). The Stockwell transform, implemented in the NAF software, uses a variable window length for the analysis which is automatically adapted along the frequency range according to the sample rate and the trial length (4^th^ order Butterworth filters with automatic padding). The time-frequency representations were normalized, separately for each condition and for each participant, by dividing each time-frequency bin by the mean power per frequency bin in a baseline period prior to the start of trials in that condition (-250 to -50 ms). This window length was also used in earlier studies [51, 72, 73, 85], since it provides a compromise between the minimum length sufficient to estimate power at the lowest frequency we report (i.e., 5Hz) and the requirement to characterise the state of the brain immediately before the onset of each trial.

To compare the time frequency representations between experimental conditions, we computed generalized linear mixed models (GLMM) using PROC MIXED in SAS (SAS Institute Inc., North Carolina, US). Time-frequency plots of percentage signal change were treated as two dimensional arrays of small time-frequency tiles, indexed in the model by three main effects, each of which is defined as a class variable: time, frequency and the interaction between time and frequency. Therefore, random effects were included in each GLMM to account for the fact that each participant’s time-frequency plot is made up of multiple time-frequency tiles. We also controlled for time-frequency (or spatial) co-variance in the spectrogram by assuming the estimates of power followed a Gaussian distribution: consequently a Gaussian link function was used in the model. The time-frequency (spatial) variability was integrated into the model by specifying an exponential spatial correlation model for the model residuals [86]. In order to account for inhomogeneity in spatial covariance in the time-frequency spectrograms, we run separate GLMMs for three broad frequency band (6–15, 15–40 and 40–50 Hz); this procedure ensure an optimal Gaussian smoothing parameter for each model. The data were resampled at a frequency resolution of 2Hz and time resolution of 25 ms, the smallest time and frequency bin consistent with model convergence. This time-frequency resolution proved optimal in other similar published studies [51, 72, 73]. Finally, we compared every full GLMM, as outlined above, with its empty equivalent model to test overall model fit. To do this we checked that there was a statistically significant reduction in -2 residual log likelihood comparing the full and empty models, as well as a substantial reduction in the Akaike Information Criterion (AIC). Both of these criteria were fulfilled for every model fitted (see S2 Table).

PROC MIXED constructs an approximate *t* test to examine the null hypothesis that the LS-Mean for percentage signal change between conditions was equal to zero in each time-frequency tile, and the procedure automatically controls for multiple comparisons (i.e. controlling experiment-wise type I error). This method has been used in multiple peer-reviewed papers (for example [51, 72, 73]). The statistical contours on the percentage signal change figures encompass time-frequency tiles fulfilling both of the following criteria: a) the difference between conditions reached *p* < 0.05; b) any region in the time-frequency plot defined by (a) also showed a response that was significantly different from zero in at least one of the two contributing conditions.

## Results

### Behavioural results

The results from the behavioural pilot are reported in S4 Fig. Participants were slower when making a superordinate as opposed to a specific level judgement, and also when categorising manmade objects. There was an interaction between these factors–the slowest responses occurred in the manmade superordinate condition, perhaps reflecting the featural diversity of manmade objects relative to animals.

During the MEG scanning, participants only responded in the case of a *mismatch* between picture and word (see Fig 1). The behavioural data confirm that participants maintained attention to the task. The overall accuracy was 84% (superordinate animal labels with manmade pictures = 82%; superordinate manmade labels with animal pictures = 88%; specific animal labels with mismatching animal pictures = 83% and specific manmade labels with mismatching manmade pictures = 83%). The percentage of false alarms was below 1% in all conditions.

### Whole brain beamforming

All of the whole-brain maps generated by this stage of the analysis can be accessed from Neurovault (http://neurovault.org/collections/1937/). Changes in oscillatory power in response to the task were seen most clearly in the frequency bands 15–25 Hz, 25–35 Hz and 35–50 Hz. In all these frequency bands, a statistically significant (p = 0.01) *reduction* in total oscillatory power was observed when the task was compared to the passive baseline period, across a widely distributed set of cortical areas linked to semantic cognition and visual processing (see Fig 2). Event related desynchronization was also observed between 5–15 Hz in right posterior fusiform gyrus, within the response observed at 15-25Hz, at a reduced threshold (p = 0.05). Power reductions in similar frequency bands have been consistently reported in studies investigating language, memory and semantic processing [51, 72, 73, 85, 87, 88], alongside power increases at higher frequencies (high gamma, > 50Hz) and at lower frequencies (theta < 5Hz), which our methods are not well-suited to investigate. Since the response to the task reflected a reduction in *total* oscillatory power across all sites and conditions, a straightforward interpretation is that the task elicited neural activity that was not phase-locked to the onset of the stimulus and/or that was variable in phase across trials and participants. Event-related *reductions* in oscillatory power, relative to oscillations at rest (especially in in a mid-frequency range from 5–30 Hz), have been linked to event-related desynchronization [89]. This type of non-phase locked response, at a similar frequency to that observed in this study, has been shown to be correlated with task-related BOLD responses in fMRI [90, 91].

Brain regions responding across conditions included (i) the anterior temporal lobes bilaterally (with a peak in anterior STG/temporal pole), (ii) the entire length of the ventral visual stream bilaterally (reaching ventral ATL), (iii) left inferior frontal gyrus extending into premotor cortex, and (iv) bilateral intraparietal sulci; cortical areas that are all known to contribute to semantic cognition (Fig 2). In addition, we observed (v) activity in right motor cortex, consistent with motor preparation for left-hand button responses, plus a small response in left central sulcus close to the motor hand area, and (vi) extensive activity in right parietal cortex which might reflect visual attention to the complex stimuli we used (picture-word combinations). These changes in oscillatory power across conditions were used to identify the locations for the POI analysis. We chose two regions of response within the ventral visual stream—ventral ATL and posterior fusiform–since these regions are thought to be important for visual object identification [45, 46]. As noted above, we also placed POIs in left central sulcus and left premotor cortex, to examine the potential motor contribution to the task.

In order to provide a more detailed report of the dataset, whole brain beamforming was also used to examine the brain’s response to the main effects of specificity (i.e., for superordinate and specific trials, relative to their own passive periods prior to these trials) and category (i.e., for animals and manmade objects). The results of this analysis are reported in S1 Fig.

### Points of Interest

**Fig 3:**
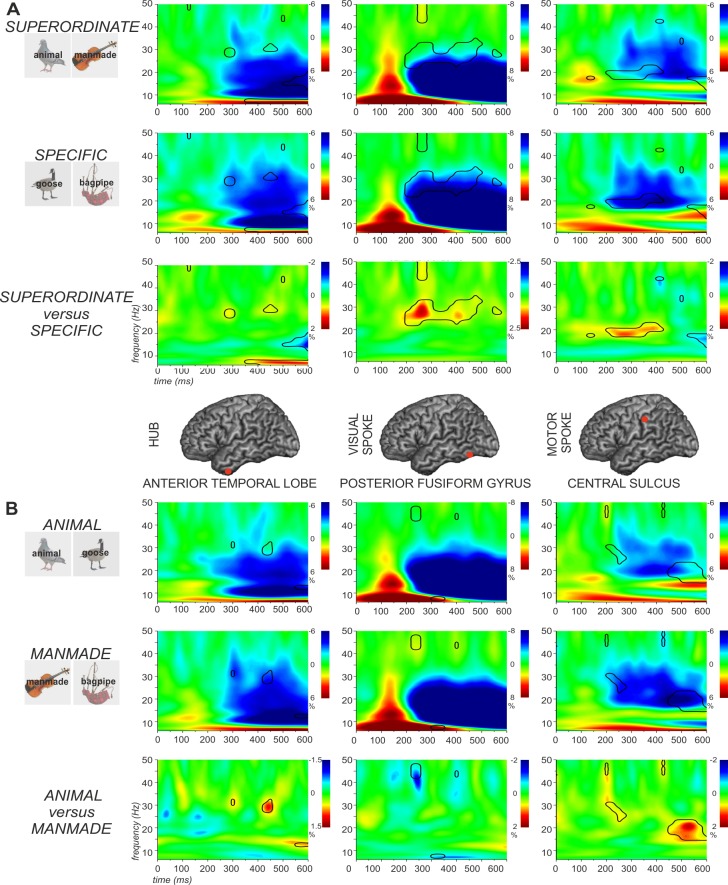
Time-frequency plots for each cortical site (ATL, FG, and CS) are presented in each column. (A) Main effect of specificity. (B) Main effect of category. In both (A) and (B), the first and second row report the percentage signal change in total power for each condition, relative to their passive periods. The third row shows differences between the two conditions. The black lines in the time-frequency plots indicate regions showing significant differences between the two conditions (p < .05). See text for details.

For each POI (ATL, FG, CS) and for each participant, we computed time-frequency (TF) plots of total power for each condition. Fig 3A shows the data for superordinate and specific judgements (i.e., the main effect of specificity). Fig 3B shows the data for animals and manmade objects (i.e., the main effect of category). The responses for each condition individually are provided in S5, S6 and S7 Figs.

Overall, for all three sites, we observed increases in power (yellow-red) relative to baseline between 50 and 150 ms (less pronounced for ATL), and decreases in power (cyan-blue) relative to baseline from 200 ms onwards. This is consistent with the possibility that power *increases* correspond to neural responses aligned to the presentation of the stimulus (characterising the brain’s response relatively early in time), while total power decreases correspond to neural activity not well-aligned to the presentation of the stimulus (potentially characterising later responses when differences between participants and trials have accumulated).

Another striking feature is the overall similarity at each site between the superordinate and specific conditions (in Fig 3A) and between the animal and manmade conditions (in Fig 3B). This suggests semantic processing arises from co-ordinated activity throughout the network, rather than, for example, particular nodes switching on and off discretely, at different points in time, or for different stimulus conditions. Nevertheless, the patterns of significant *differences* between conditions at each site (bottom row in Fig 3A and 3B) also suggest that, superimposed on this co-ordinated network activity, is a pattern of stimulus- and task-specific differences that arise as a result of varying the relative strength of the contributions from different nodes at different points in time.

For the main effect of specificity, the comparison of superordinate and specific conditions revealed stronger power reductions for specific judgements in the beta and low gamma frequency bands in all three POIs (Fig 3A). In ATL, task-related reductions in oscillatory power extended into higher frequencies (25–35 Hz) for specific judgements compared with superordinate judgements from 300–500 ms post-target onset. There was a similar effect of specificity in posterior fusiform cortex, with a stronger response for specific trials from 200–500 ms, as well as for superordinate judgements between 250–300 ms at 40–50 Hz. In the central sulcus, there was a stronger task-related power decrease in the specific condition from 15–25 Hz, extending in time from 200–400 ms, plus a stronger power increase for superordinate-level matching in the first 200 ms. Thus, effects of specific > general on the strength of task-related power decreases were striking from around 200 ms across sites.

**Fig 4:**
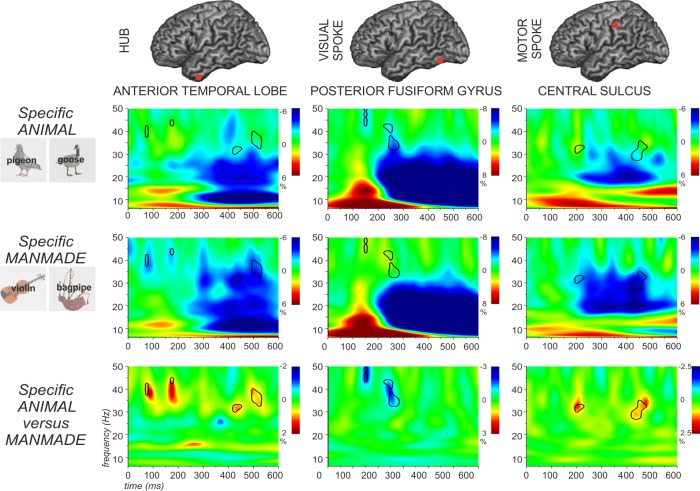
Percentage signal change in total power for animal and manmade objects judgements at the specific level are reported in the first and the second row. The third row shows differences between the two conditions. The black lines in the time-frequency plots indicate regions showing significant differences between the two conditions (p < .05). Each cortical site (ATL, FG, and CS) is presented in each column. See text for details.

With respect to the main effect of category, the comparison of animals and manmade objects revealed earlier and stronger power reductions for the manmade category in the central sulcus as predicted, from 200–600 ms in the beta and low gamma bands (Fig 3B). Stronger power changes in low gamma starting from around 250 ms were also observed in fusiform cortex and ATL for manmade objects compared with animals. Fig 4 shows the effect of category at the specific level (i.e., pigeon *versus* guitar). As noted in the Introduction, we would expect to see larger category effects in posterior fusiform cortex for specific-level trials, since the distinctive visual properties of animals are thought to be important for distinguishing between these items that generally share many features. Consistent with this prediction, posterior fusiform cortex showed greater task-related decreases in oscillatory power for the specific animal condition contrasted with the specific manmade condition (100–200 ms, 30–50 Hz). The reverse pattern was observed in central sulcus: the specific manmade condition showed a stronger response at this site (200 and 400 ms at 30 Hz). Consequently, across these visual and motor sites, differences between animals and manmade objects were in opposite directions, with stronger power reductions for animals compared with manmade objects in fusiform cortex, and stronger power reductions for the manmade than the animal condition in central sulcus. Finally, there were stronger power reductions in left ATL for tools than for animals from around 100 ms post-onset at 40 Hz, which persisted until around 550 ms (Fig 4).

We also assessed the possibility of an interaction between specificity and category at each site (see S5, S6 and S7 Figs). For this analysis, we first examined the specificity effect for each category separately and then compared these effects (i.e. a difference of differences) in order to establish if there was a larger effect of specificity for one category compared with another. In ATL, there were specificity effects for *both* categories (greater reductions in total oscillatory power for specific identification), and this effect was stronger for manmade objects briefly at around 500 ms and 30 Hz. Left posterior fusiform cortex (visual site) also showed an effect of specificity for both animals and manmade objects: again, this effect resulted in greater decreases in total oscillatory power, and this response was stronger for animals in the first 200 ms above 40 Hz, consistent with the prediction that visual processes are particularly important in distinguishing between animals which have highly-overlapping visual features at the specific level. The left posterior fusiform POI also showed a stronger contribution to the identification of manmade objects at general level, as shown by the ‘reverse specificity effect’ for manmade objects observed in Figs 3A and 4 and S6 Fig (this time reflected in greater power increases relative to baseline for general judgements). This effect could conceivably reflect the greater visual diversity of manmade objects, increasing the contribution of the visual system to their identification at a general level. The central sulcus showed a complex pattern of response: although this site showed a main effect of category (manmade > animal concepts) and a main effect of specificity (specific > superordinate), it also showed a ‘reverse specificity effect’, with a stronger response in the superordinate compared to the specific condition for the animal category at 35 Hz and for the manmade category at 20 Hz around 500 ms post-onset. This elicited a significant interaction.

## Discussion

This study used MEG to explore how conceptual retrieval emerges from a distributed network comprising the ATL ‘hub’ plus putative sensory/motor ‘spokes’ in fusiform gyrus (visual) and central sulcus (somatomotor). In a word-picture verification task involving items from two semantic categories (animals and manmade tools) and matching at two levels of specificity (using superordinate labels like ‘animal’ and more specific terms like ‘pigeon’), we found engagement across all conditions at all sites and yet also differences in the strength of these responses across conditions and categories. ATL showed sensitivity to both category and specificity: early transient differences between conditions were seen within 100 ms (outside the main region of response to stimulus presentation in the time-frequency plots), which were followed by further significant differences between conditions from around 300 ms to the end of the analysis window. This was the case for the overall contrast between specific and superordinate judgements, and also for the comparison between manmade objects and animals at the specific level. We also found a double dissociation between visual and motor ‘spoke’ regions for specific-level judgements, consistent with our predictions: a visual site within posterior lateral fusiform showed event-related power reductions that were stronger for animals than for manmade objects during specific identification from 150–250 ms, while a motor site in central sulcus showed a larger response to manmade objects than animals from 200 ms. Thus, our work provides evidence that conceptual identification draws on both an ATL ‘hub’ and visual and motor ‘spokes’, with the engagement of these sites being determined by the level of identification required and the relevance of each sensory-motor feature to the concept presented on that trial.

There were at least two components of the response that followed the presentation of a visual stimulus. First, in the motor and visual ‘spoke’ sites, and to a lesser extent in ATL, there were transient *increases* in oscillatory power particularly in the alpha band (shown in red in the total power plots for each condition relative to the passive period): this response occurred from about 100 ms post-stimulus onset, and was mostly insensitive to specificity and category (if anything, this response was actually stronger for superordinate matching trials, at least in the motor spoke; see Fig 3). Secondly, there were more sustained *decreases* in oscillatory power relative to baseline, largely in beta and low gamma frequencies, which were observed from around 200 ms post-stimulus. This may have reflected the recruitment of larger numbers of neurons firing asynchronously when greater feature retrieval was required. Our observation of more substantial power reductions when sites were expected to be making a greater contribution to semantic processing is consistent with the account of Hanslmayr et al. [89], who showed that local desynchronization would allow more information to be maintained and processed. Furthermore, EEG power decreases in alpha and beta frequency bands have been linked to successful encoding and episodic memory retrieval and the reactivation of sensory features of memory traces during successful retrieval [92–94], consistent with the category-specific effects we observed.

These findings help to refine the ‘hub and spoke’ framework for semantic representation, implemented as a computational model by Rogers et al. [4, 95]. According to this account, conceptual representations are acquired by an amodal ‘hub’ in ATL which interacts with modality-specific sensory and motor features. This allows the ATL to compute deep *conceptual* similarities that are not strongly influenced by the superficial similarity between two concepts in a particular modality: for example, pear and light bulb have similar shapes but the ATL instead captures the greater conceptual overlap between pear and pineapple. The pattern of activation across the ATL units is thought to be highly overlapping for semantically-related concepts that share many features *across* modalities (e.g., horse and zebra) but distinct for concepts drawn from different categories (e.g., horse and screwdriver). Thus, the ATL is expected to show a greater response when specific concepts must be identified, since this involves being able to distinguish between the target concept and highly similar patterns of activation that represent other items in the same category. Neuroimaging studies have also shown more ATL recruitment for specific-level identification [15, 36, 48], while inhibitory TMS applied to this area disrupts picture naming at the specific-level more than the superordinate-level [96]. Related to this pattern, a recent MEG study found a greater response in ATL at ~250 ms for adjective-noun phrases, for example ‘red boat’, compared to when the noun was preceded by a non-word (e.g. xhl) or a control word that could not be combined with the noun (e.g. cup) [47, 48]. This combinatorial response might similarly reflect a greater role for ATL in retrieving specific concepts specified by combinations of words [97, 98]. Our findings confirm this pattern in ATL beyond combinatorial linguistic stimuli using superordinate vs. specific levels of word-picture matching.

In addition to effects of specificity within ATL, our data revealed effects of specificity within the spokes, which were category-dependent. Event-related power decreases in a visual site in left posterior fusiform cortex were stronger during the identification of animals at the specific-level, while a motor site in central sulcus showed stronger event-related power decreases for manmade objects. Animals generally share many visual features (e.g., four legs, eyes etc.) and thus the identification of animal concepts at the specific-level is thought to require a greater contribution from visual cortex to allow for the discrimination between overlapping representations [4]: e.g., the visual feature “stripy”‘ is critical to distinguishing a zebra from a horse. The posterior fusiform site also showed increased total oscillatory power during the superordinate categorisation of manmade objects: this might reflect the fact that manmade objects are more diverse and tend to have more unique visual features than animals; consequently, the identification of these objects at a superordinate level may require greater work within the visual system than the superordinate identification of animals [18, 19, 99, 100]. In addition, manipulable manmade objects have more prominent action features than animals and thus the identification of tools might require greater engagement of motor cortex (and other brain regions coding for visual motion and hand praxis) in concert with ATL [21, 101–109]. These findings are broadly compatible with neuroimaging and lesion studies which suggest a greater involvement of visual areas (especially lateral fusiform) in the identification of animals, and of fronto-parietal action and praxis areas for tools [77, 110–114].

In recent years, time-sensitive imaging methods have started to explore interactions between the ATL hub and visual processes in object recognition. These studies suggest that the retrieval of a coarse-grained semantic representation occurs throughout the feed-forward propagation of activity along ventral temporal cortex [44–46, 115] and that recurrent mechanisms within the same network support the retrieval of more detailed representations [45, 51, 116]. For example, in a study of picture naming [46], evoked MEG responses in ATL and posterior fusiform cortex were first sensitive to the number of shared features among the stimuli (~100 ms), supporting coarse-grained identification, and then to distinctive features (~230 ms). While these studies have focussed on the contribution of both anterior and posterior temporal areas to visual object recognition, the current study shows that motor features may make a similar contribution to the identification of tools. Thus interactions between ATL and visual cortex identified in previous studies might be an example of hub-and-spoke processing that characterises specific semantic retrieval in the brain more widely.

Category effects were also observed in the ATL hub: there were early and sustained changes in total power for both categories but these were more pronounced for manmade objects compared to animals. Category effects were not predicted for ATL, since semantic dementia patients with atrophy and hypometabolism centred on this region rarely show category-specific semantic deficits once concept familiarity is controlled for [117]. Indeed, the literature tends to show either no difference between animals and manmade objects (in lateral ATL [36, 38, 118, 119]), or a stronger involvement of ATL for animals (in medial aspects)–for example, semantic deficits in patients with herpes simplex encephalitis are often greater for animals and these cases have more medial damage than in semantic dementia [19, 110, 120]. Indeed, fMRI, MEG and intracranial recording studies have all revealed a stronger response to animals compared with manmade objects in medial aspects of ATL [45, 49, 100, 118, 121, 122]. Medial ATL is thought to play a critical role in fine-grained visual identification (critical for distinguishing between different animals); however, it appears that our results are not a consequence of our region-of-interest location, since a second virtual electrode in medial ATL showed the same pattern (manmade > animal; see S3 Fig). One potential way of reconciling the literature with our pattern of findings is to note that although there are regions in the time-frequency plot that show manmade > animal effects, there was not a stronger response in ATL to manmade objects *overall* in our data: instead, we found this effect transiently at a specific frequency band (30–40 Hz). Taken together, these results appear to be consistent with the view that ATL supports the identification of both animals and manmade objects–yet the oscillatory response to animals and manmade objects may be different, for example reflecting different patterns of communication with ‘spoke’ regions [21]. For example, our results might reflect greater within-hemisphere connectivity between the ATL and regions engaged in action understanding, which recruits a left-lateralised network [12, 123].

The effects of category in the ATL emerged within 100 ms of stimulus presentation; these effects were not clearly contiguous with the core event-related power decrease seen across conditions, but preceded this general response. Previous electrophysiological studies have also reported lexical-semantic processing effects in left anterior temporal regions before 200 ms [45, 48, 51] and sensitivity to gross category within 100 ms [49, 115, 124, 125]. What might these early and transient responses within ATL reflect? While it seems unlikely that there could be full instantiation of target concepts this rapidly (instead, the responses in both hub and spokes from 250 ms are more likely to correspond to such a process), coarsely-coded visual input might enable predictions to be made about the likely stimulus category and task requirements of the trial, which would allow appropriate processing pathways to be established within the hub and spokes semantic network. Early category effects in ATL might reflect sensitivity to the shared gross visual characteristics of living and manmade objects, allowing differential engagement of the relevant set of spokes. Similarly, a differential response to superordinate and specific trials in this experiment could have been achieved through sensitivity to whether the orthographic input corresponded to either of the two superordinate-level labels (i.e., the words ‘animal’ and ‘manmade’), which were repeated across many trials, or to whether the printed word was not identical to many previous inputs (suggesting specific level identification would be required).

In conclusion, the MEG data presented here provide support for a model of semantic retrieval involving an ATL ‘hub’ and motor and sensory ‘spokes’. Following the visual presentation of an object for identification, there is (i) an initial ‘flash’ of activation through the system, which is largely insensitive to category/specificity; (ii) early differential responses in ATL that reflect category and specificity–and which might allow an appropriate broader network to be recruited in a way that reflects the demands of the task and stimulus; and (iii) sustained engagement of the ATL hub and spokes, with a dissociation between the spokes reflecting greater engagement of motor representations for tools and greater engagement of lateral fusiform visual processes for animals when concepts are identified at the specific level. Overall, these findings are compatible with a model of semantic cognition in which conceptual identification emerges from the simultaneous recruitment of hub and spoke sites, as opposed to the extraction of feature knowledge in spoke sites which precedes conceptual identification in the ATL hub.

## Supporting Information

S1 Fig Whole brain beamforming: main effects of specificity and category. (PDF)

S2 Fig Additional Point of Interest in the Premotor Cortex. (PDF)

S3 Fig Additional ATL site. (PDF)

S4 Fig Behavioural Results. (PDF)

S5 Fig Interaction between specificity and category effects in Anterior Temporal Lobe. (PDF)

S6 Fig Interaction between specificity and category effects in Fusiform Gyrus. (PDF)

S7 Fig Interaction between specificity and category effects in Central Sulcus. (PDF)

S1 Table Figure and statistics of the properties of the stimuli. (PDF)

S2 Table Summary of test of the model fit. (PDF)
