# Supplementary material for: Oscillatory Dynamics Supporting Semantic Cognition: MEG Evidence for the Contribution of the Anterior Temporal Lobe Hub and Modality-Specific Spokes
Source: PLoS One. 2017 Jan 11;12(1):e0169269. doi: 10.1371/journal.pone.0169269 (PMC5226830; doi:10.1371/journal.pone.0169269)
Supplement: S1 Fig — (PDF) [file pone.0169269.s001.pdf]

**Whole brain beamforming: main effects of specificity and category.**

Whole brain beamforming was used to examine the brain's response to Specific and Superordinate trials, and to Animal and Manmade trials. This showed that animals and manmade objects produced a similar response in bilateral fusiform and lateral occipital cortex. There was also a response for both categories in anterior temporal and lateral frontal regions. This frontal response was somewhat more marked in the manmade condition, although significant category differences were not found in statistical comparisons of these maps. The response in all these regions was also apparent in both general and specific trials: the response in the left-hemisphere fusiform and lateral occipital trials was somewhat more marked in specific than superordinate trials (but again significant differences were not seen in whole-brain comparisons).

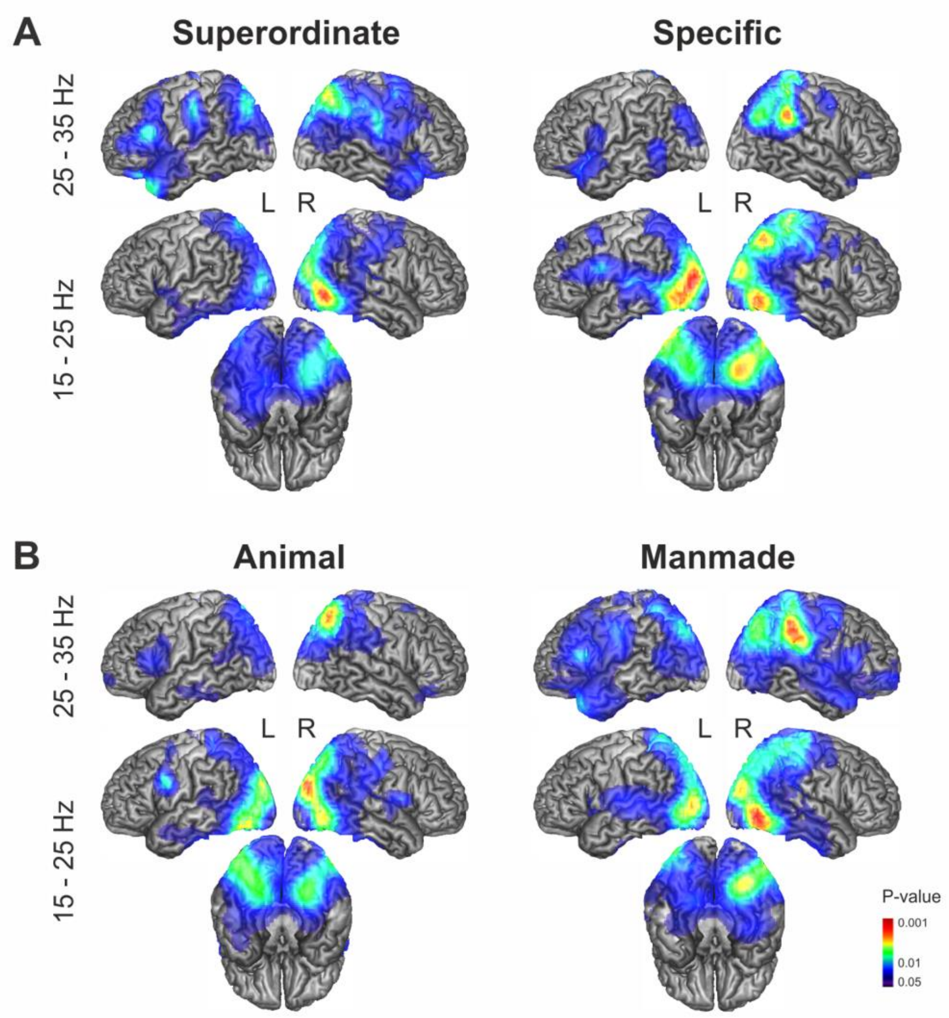

**S1 Fig. Whole brain beamforming: main effects of specificity and category.**

Whole-brain images showing neural activity above baseline for the main effects of specificity (A) and category (B), during 500 ms post-target onset in four frequency bands (15-25 Hz, 25-35 Hz and 35-50 Hz). t-Maps are thresholded at $p$<0.05 (corrected). All the activations represent event related desynchronization.
