# Supplementary material for: Oscillatory Dynamics Supporting Semantic Cognition: MEG Evidence for the Contribution of the Anterior Temporal Lobe Hub and Modality-Specific Spokes
Source: PLoS One. 2017 Jan 11;12(1):e0169269. doi: 10.1371/journal.pone.0169269 (PMC5226830; doi:10.1371/journal.pone.0169269)
Supplement: S2 Fig — (PDF) [file pone.0169269.s002.pdf]

Additional Point of Interest in the Premotor Cortex

An addition motor site was identified in the premotor cortex (PMC). Previous literature has reported this region as associated with tool and action comprehension [1-4].

In all the conditions, power increase relative to the baseline was observed in alpha and beta bands within the first 250ms and at lower frequencies throughout the entire window. Starting from 200ms, decrease in total power was reported in beta and low gamma bands.

The comparisons between general and specific judgements revealed a late 'reverse specificity effect' showing a stronger response to general judgements after 400ms (S2A Fig). Starting from 200ms, a main effect of category was also observed with a stronger response to manmade object compared to animals (S2B Fig). Similarly, a category effect at specific level revealed stronger power reduction for specific tools in low gamma band after 300ms from stimulus onset (S2C Fig).

Although part of the response in this site might be contaminated by semantic control processes [5], the pattern of results observed in the PMC are broadly consistent with the effects observed in the CS.

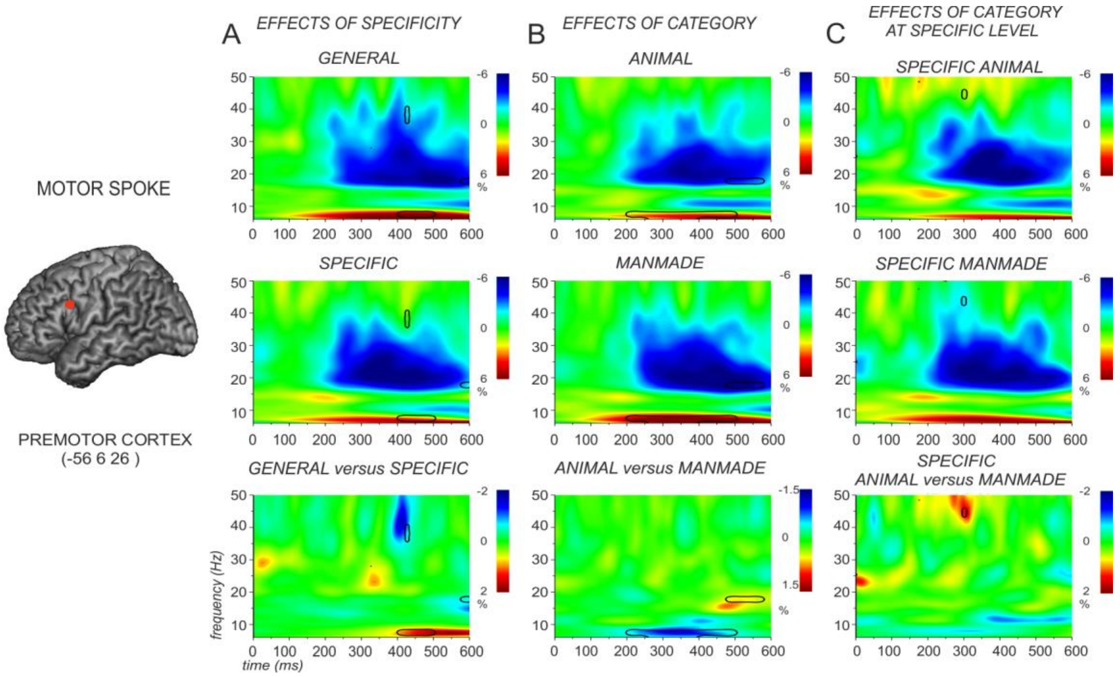

S2 Fig. Additional Point of Interest in the Premotor Cortex. Time-frequency plots in the Premotor Cortex. (A) and (B) illustrate the main effects of specificity and category,

respectively; (C) reports data for animal and manmade objects judgements at the specific level. The first and the second rows reports the percentage signal change in total power for each condition relative to their passive periods. The third row shows differences between the two conditions. The black lines in the time-frequency plots indicate regions showing significant differences between the two conditions (p<.05).

References

1. Rueschemeyer, S.-A., et al., *The function of words: distinct neural correlates for words denoting differently manipulable objects.* Journal of Cognitive Neuroscience, 2010. **22**(8): p. 1844-51.
2. Chao, L.L., J. Weisberg, and A. Martin, *Experience-dependent modulation of category-related cortical activity.* Cereb Cortex, 2002. **12**(5): p. 545-551.
3. Ishibashi, R., et al., *The neural network for tool-related cognition: An activation likelihood estimation meta-analysis of 49 neuroimaging studies.* Cognitive Neuropsychology, 2016. **33**(3-4): p. 241-56.
4. Liljestrom, M., et al., *Perceiving and naming actions and objects.* Neuroimage, 2008. **41**(3): p. 1132-41.
5. Noonan, K.A., et al., *Going beyond inferior prefrontal involvement in semantic control: evidence for the additional contribution of dorsal angular gyrus and posterior middle temporal cortex.* J Cogn Neurosci, 2013. **25**(11): p. 1824-50.
