# Supplementary material for: Oscillatory Dynamics Supporting Semantic Cognition: MEG Evidence for the Contribution of the Anterior Temporal Lobe Hub and Modality-Specific Spokes
Source: PLoS One. 2017 Jan 11;12(1):e0169269. doi: 10.1371/journal.pone.0169269 (PMC5226830; doi:10.1371/journal.pone.0169269)
Supplement: S3 Fig — (PDF) [file pone.0169269.s003.pdf]

Additional ATL site.

As noted in the main body, we were unable to identify a local peak in ventral ATL because the response was strongest in visual cortex and progressively reduced in strength from posterior to anterior temporal areas. We therefore examined an ROI based on findings in the fMRI literature on semantic processing (MNI coordinate: -22,-6,-20). In order to confirm that similar effects would be observed in other ATL locations implicated in semantic processing, time frequency analysis was also performed on a set of coordinates implicated in visual object recognition [1]. The location of this ROI is more ventral and medial to the site reported in the main text.

The time-frequency results from this additional ATL site are largely consistent with the analysis reported in the main body. Power increases for the word-picture matching task relative to the baseline were observed at low frequencies throughout the entire time window and also in alpha and beta bands within the first 200ms; whereas reductions in total power relative to rest characterized the neural activity of the six conditions in beta and low gamma bands.

Except for a brief strong response for general judgement after 150ms at 45Hz, these power decreases were stronger for specific compared to general judgements (S3A Fig); this latter pattern in time frequency space resembles the significant effects reported for the other ATL site. Such similarities are also observed for the category effects (S3B Fig). Finally, the comparison of animal and tools at specific level revealed stronger power reduction for tools in beta and low gamma band starting around 200ms, but becoming significant after 400ms (S3C Fig).

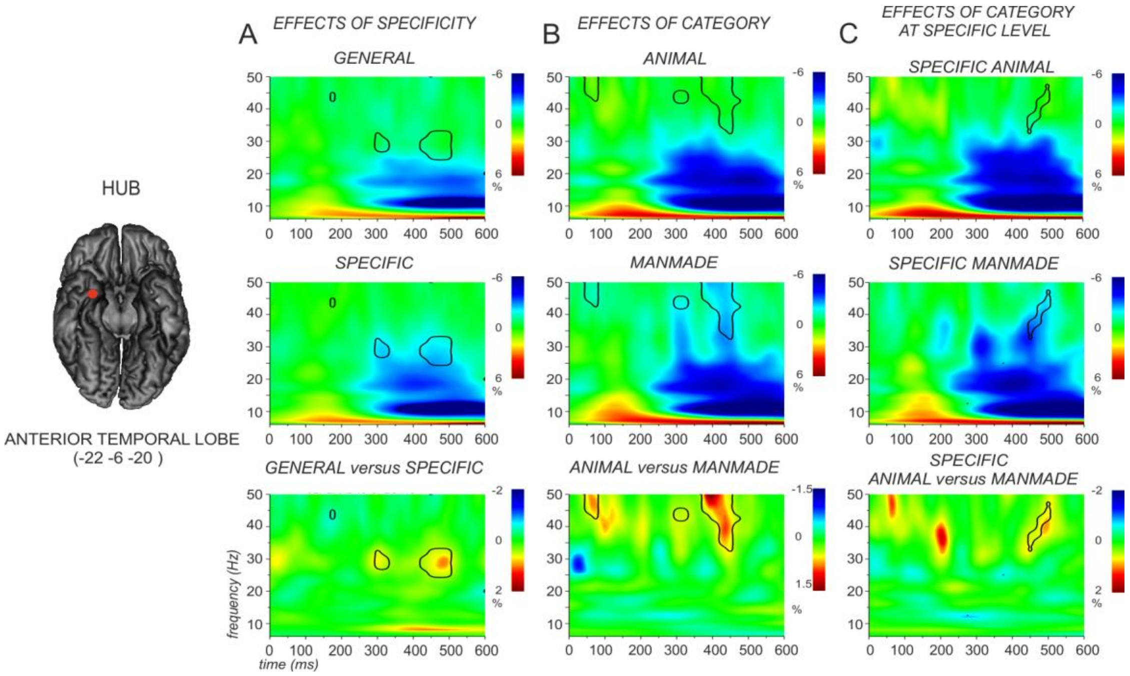

S3 Fig. Additional ATL site. Time-frequency plots for the ventro-medial ATL site. (A) and (B) illustrate the main effects of specificity and category, respectively; (C) reports data for animal and manmade objects judgements at the specific level. The first and the second rows reports the percentage signal change in total power for each condition relative to their passive periods. The third row shows differences between the two conditions. The black lines in the time-frequency plots indicate regions showing significant differences between the two conditions (p<.05).

References

1. Clarke, A., K.I. Taylor, and L.K. Tyler, *The evolution of meaning: spatio-temporal dynamics of visual object recognition.* Journal of Cognitive Neuroscience, 2010. **23**(8): p. 1887-99.
