# Supplementary material for: Oscillatory Dynamics Supporting Semantic Cognition: MEG Evidence for the Contribution of the Anterior Temporal Lobe Hub and Modality-Specific Spokes
Source: PLoS One. 2017 Jan 11;12(1):e0169269. doi: 10.1371/journal.pone.0169269 (PMC5226830; doi:10.1371/journal.pone.0169269)
Supplement: S4 Fig — (PDF) [file pone.0169269.s004.pdf]

Behavioural Results

The experimental setup used in the MEG experiment was tested outside the scanner. In this pilot experiment, participants responded to both match and mismatch trials by pressing one of two mouse buttons to indicate if the word corresponded with the picture. The analysis below focuses on correct response times to "match" trials since on these trials, it is possible to categorise processing in terms of both specificity and category (i.e., the category of the word matched the picture). Participants' performance was evaluated using a response efficiency measure, computed dividing the RT by the proportion of correct answers. These data were analysed using repeated-measures ANOVA with level of *specificity* (superordinate - SO, specific - S) and *category* (animal - A, manmade - M) as within-subjects factors. This revealed main effects of *specificity* ($F_{(1,15)}$= 23,67, p <0.001) and *category* ($F_{(1,15)}$= 69,82, p <0.001). Responses were more efficient in the specific condition than the superordinate condition, perhaps because more of these items were basic-level terms which are typically retrieved faster than superordinate level terms [31]. There was also a significant interaction between category and specificity ($F_{(1,15)}$= 37,84, p <0.001). Bonferroni-corrected post-hoc comparisons revealed that participants' efficiency for Superordinate Manmade (SOM) trials was significantly lower than the other conditions (SOM *vs* SOA: $t_{(15)}$= 8.48, p <0.001; SOM *vs* SA: $t_{(15)}$= 8.37, p <0.001; SOM *vs* SM: $t_{(15)}$= 7.48, p <0.001). This might reflect the difficulty of superordinate matching given the lower featural overlap for manmade objects compared to animals [108].

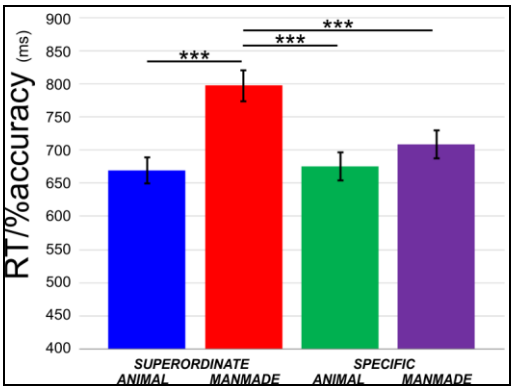

S4 Fig. Behavioural Results. Mean efficiency scores to the 'match' trials per condition. Error bars represent standard error of the mean.*** p <0.001.
