# Supplementary material for: Oscillatory Dynamics Supporting Semantic Cognition: MEG Evidence for the Contribution of the Anterior Temporal Lobe Hub and Modality-Specific Spokes
Source: PLoS One. 2017 Jan 11;12(1):e0169269. doi: 10.1371/journal.pone.0169269 (PMC5226830; doi:10.1371/journal.pone.0169269)
Supplement: S6 Fig — (PDF) [file pone.0169269.s006.pdf]

Interaction between specificity and category effects in Fusiform Gyrus.

This analysis shows effects of specificity for both categories, plus an interaction that reflects greater effects of specificity for animals than manmade objects.

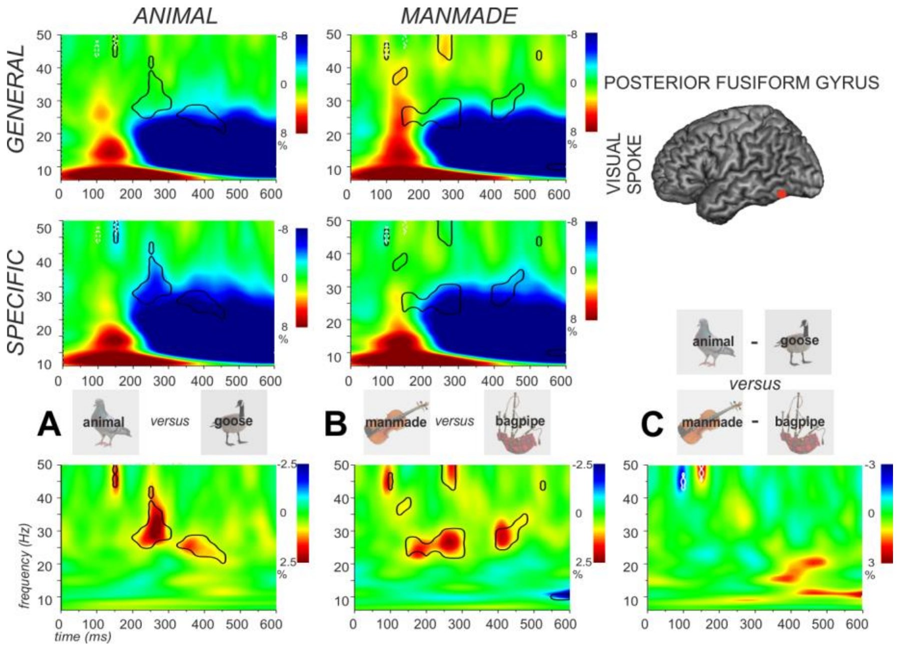

S6 Fig. Interaction between specificity and category effects in Fusiform Gyrus.

The first two rows show the percentage signal change in total power for each condition relative to the passive period for the same trials. (A) and (B) report the effects of specificity for animal and manmade objects separately. The black lines in the time-frequency plots indicate regions showing significant specificity differences between the two conditions (for animal and manmade trials separately) that fulfilled both of the following criteria: a) the difference between conditions reached $p < 0.05$; b) any region in the time-frequency plot defined by (a) was also significantly different from the baseline in at least one of the two contributing conditions. (C) illustrates the interaction between specificity and category. Significant interactions – i.e., differences in the effect of specificity for animal and manmade trials – are shown by white lines. These lines enclose regions of the time-frequency plot fulfilling both of the following criteria: a) the difference between these specificity contrasts reached $p < 0.05$; b) any region in the time-frequency plot defined by (a) was also significant in at least one of the two contributing analyses of specificity. To help with the interpretation of the interaction results, the areas of difference between the effects of specificity for each

category are also indicated by white dashed lines in the time-frequency plot of each condition. See the main text for a summary of these results.
