# Supplementary material for: Oscillatory Dynamics Supporting Semantic Cognition: MEG Evidence for the Contribution of the Anterior Temporal Lobe Hub and Modality-Specific Spokes
Source: PLoS One. 2017 Jan 11;12(1):e0169269. doi: 10.1371/journal.pone.0169269 (PMC5226830; doi:10.1371/journal.pone.0169269)
Supplement: S1 Table — (PDF) [file pone.0169269.s008.pdf]

S1 Table. Figure and statistics of the properties of the stimuli.

	CONDITION	MEAN (sd)	t	Sig.(2-tailed)
NUMBER OF LETTERS	ManMade *	6.1 (1.7)	-0.21	0.84
	Animal*	6.2 (1.8)		
LEXICAL FREQUENCY	ManMade	2161.3 (5868.7)	-0.38	0.71
	Animal	2497.8 (3744.8)		
FAMILIARITY	ManMade	3.8 (.6)	1.22	0.23
	Animal	3.7 (.5)		
IMAGE AGREEMENT	ManMade	4.5 (.2)	-1.28	0.2
	Animal	4.5 (.2)		
NUMBER OF NON-WHITE PIXEL	ManMade	11755.7 (6140.8)	-0.37	0.72
	Animal	12112.5 (4403.5)		
TYPICALITY	ManMade	3.86 (.77)	-2.63	0.01
	Animal	4.18 (.53)		
NAME AGREEMENT	ManMade	18.08 (2.94)	-0.94	0.35
	Animal	18.52 (2.04)		
PERCEPTUAL FEATURES				
Visual	ManMade	3.54 (.32)	-11.55	<.001
	Animal	4.24 (.34)		
Action	ManMade	3.28 (.55)	18.64	<.001
	Animal	1.86 (.21)		
Visual-Motion	ManMade	2.09 (.48)	-7.6	<.001
	Animal	2.67 (.33)		

*Manmade Objects: musical instruments, household items, tools, kitchen utensils, weapons

*Animal: insects, birds, mammals, fishes and shellfishes
