# Supplementary material for: Oscillatory Dynamics Supporting Semantic Cognition: MEG Evidence for the Contribution of the Anterior Temporal Lobe Hub and Modality-Specific Spokes
Source: PLoS One. 2017 Jan 11;12(1):e0169269. doi: 10.1371/journal.pone.0169269 (PMC5226830; doi:10.1371/journal.pone.0169269)
Supplement: S2 Table — (PDF) [file pone.0169269.s009.pdf]

S2 Table. Summary of test of the model fit.

The overall model fit was tested by comparing the '-2 residual log likelihood' and the 'Akaike Information Criterion' (AIC) of the full model and an empty model.

POI	Frequency band / Fit statistic	Specificity		Category		Category v Specific	
		Full	Empty	Full	Empty	Full	Empty
LATL	6-15Hz						
	-2 Log Likelihood	10227.1	11047.4	10529.6	11175	12033.2	12631.3
	AIC	10231.1	11049.4	10533.6	11177	12037.2	12633.3
	15-40Hz						
	-2 Log Likelihood	15698.5	24124	15107.4	23220.2	18448.4	26959.8
	AIC	15702.5	24126	15111.4	23222.2	18452.4	26961.8
	40-50Hz						
	-2 Log Likelihood	3535.7	10298.8	3549.4	10434	5225.1	12224.1
	AIC	3539.7	10300.8	3553.4	10436	5229.1	12226.1
FG	6-15Hz	10562.4	11244.4	11100.1	11814.7	12456.6	13194.3
	-2 Log Likelihood	10566.4	11246.4	11104.1	11816.7	12460.6	13196.3
	AIC						
	15-40Hz	16323	25283.9	15997.6	24782.4	19538.3	28256.6
	-2 Log Likelihood	16327	25285.9	16001.6	24784.4	19542.3	28258.6
	AIC						
	40-50Hz	3444.7	10328.8	3650.3	10756.7	4892.2	12046.6
	-2 Log Likelihood	3448.7	10330.8	3654.3	10758.7	4896.2	12048.6
	AIC						
PC	6-15Hz	10617.5	11285.7	10975.1	11725.9	12214	12691.8
	-2 Log Likelihood	10621.5	11287.7	10979.1	11727.9	12218	12693.8
	AIC						
	15-40Hz	16027.6	25021.8	16698.4	25624.4	19967.5	28706.9
	-2 Log Likelihood	16031.6	25023.8	16702.4	25626.4	19971.5	28708.9
	AIC						
	40-50Hz	3821.3	10738.8	3907.8	10836.6	5435.5	12584.7
	-2 Log Likelihood	3825.3	10740.8	3911.8	10838.6	5439.5	12586.7
	AIC						
